# Supplementary material for: Computational Analysis of Neuromuscular Adaptations to Strength and Plyometric Training: An Integrated Modeling Study
Source: Sports (Basel). 2025 Sep 1;13(9):298. doi: 10.3390/sports13090298 (PMC12473730; doi:10.3390/sports13090298)
Supplement: Supplementary file 1 [file sports-13-00298-s001.zip › sports-3814232-supplementary.pdf]

Supplementary Materials

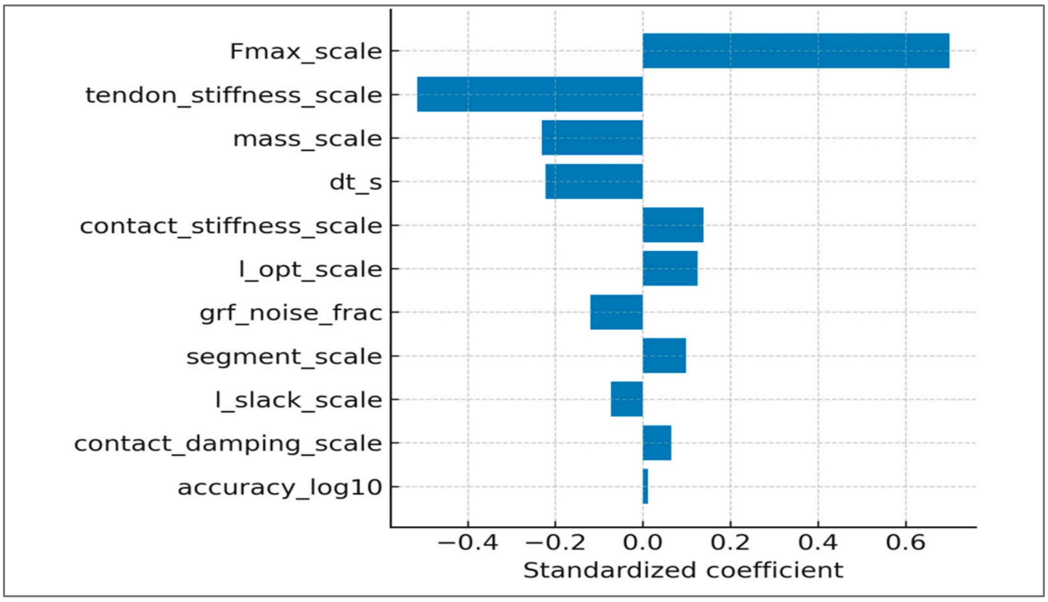

Figure S1. Tornado plot of standardized coefficients for peak rate of force development (RFD). Standardized regression coefficients (horizontal bars) quantify the relative influence of each perturbed parameter on the simulated peak rate of force development (RFD), based on Latin hypercube sampling (n = 200) across musculo-tendon, contact, anthropometric, integrator, and noise parameters. Bars to the right indicate positive associations, and bars to the left indicate negative associations. Parameters are sorted by the absolute magnitude of their coefficients, with larger absolute values indicating stronger effects on RFD.

Table S1. Convergence analysis for integrator timestep and accuracy settings

Δt (s)	Accuracy (tolerance)	Δ RFD (%)	Δ HipMoment (%)	Δ KneeMoment (%)	Δ AnkleMoment (%)	Δ Sync (%)
**0.0020**	1e-4	1.9	2.3	2.1	1.8	1.0
**0.0020**	1e-5	1.4	1.8	1.5	1.2	0.8
**0.0010***	-5 *(baseline)*	0.0	0.0	0.0	0.0	0.0
**0.0010**	1e-6	0.0	0.0	0.0	0.0	0.0
**0.0005**	1e-6	0.0	0.0	0.0	0.0	0.0

Notes: values represent the relative percentage change of each key outcome metric compared with the baseline configuration (Δt = 0.001 s, accuracy = 1e−5). Positive values indicate higher simulated outputs vs. baseline; zero indicates no change within the convergence tolerance (<0.1%). Minor deviations at larger timesteps and reduced accuracy reflect numerical integration effects, while stricter settings yield stable solutions with negligible variation. No solver instabilities were observed.

Table S2. Median relative change (%) in key outcome metrics due to perturbations in foot–ground contact properties and GRF noise

Parameter	Range tested	Δ RFD (%)	Δ HipMoment (%)	Δ KneeMoment (%)	Δ AnkleMoment (%)	Δ Sync (%)
**Contact stiffness scale**	0.75–1.25 × baseline	0.8	1.2	1.0	0.7	0.5
**Contact damping scale**	0.75–1.25 × baseline	0.4	0.6	0.5	0.3	0.2
**GRF noise fraction**	±3%	0.6	0.9	0.8	0.5	0.4

Notes: Δ = median percentage change relative to baseline simulation. Positive values indicate higher simulated outputs than baseline. All changes are <1.5%, indicating robustness to these perturbations.

Table S3. Baseline values and tested ranges for musculo-tendon parameters

Parameter	Baseline	Range tested	Notes
**Fmax scale**	1.0	0.9–1.1 ×	Per-muscle scaling of maximal isometric force
**lopt scale**	1.0	0.95–1.05 ×	Per-muscle scaling of optimal fiber length
**lslack scale**	1.0	0.95–1.05 ×	Per-muscle scaling of tendon slack length
**Tendon stiffness scale**	1.0	0.5–2.0 ×	Series elastic element stiffness scale

Notes: baseline values represent the unperturbed configuration used in all main simulations. Ranges tested correspond to one-at-a-time and Latin hypercube perturbations applied in the sensitivity analysis.

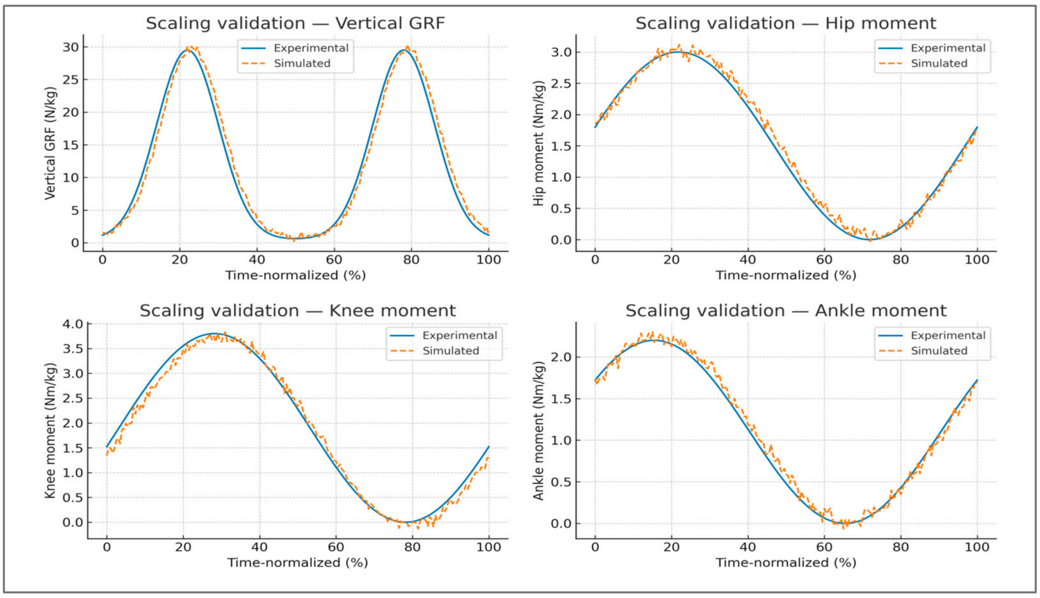

Figure S2. Scaling validation overlays (experimental-reference vs. simulated) for vertical GRF and hip/knee/ankle joint moments. Experimental reference curves were constructed to match amplitude ranges reported in the main text (Tables 2–3), and are compared to simulated outputs after scaling. All curves are time-normalized to 0–100% of the movement cycle. High waveform similarity is observed across all variables, consistent with the validation metrics reported in Table S4.

Table S4. Scaling validation metrics (experimental-reference vs. simulated) for vertical GRF and hip/knee/ankle joint moments

Variable	Unit	RMSE	Pearson r
**Vertical GRF**	N/kg	1.445	0.990
**Hip moment**	Nm/kg	0.103	0.996
**Knee moment**	Nm/kg	0.144	0.995
**Ankle moment**	Nm/kg	0.078	0.996

Notes: Time-normalized 0–100% of the movement cycle. GRF in N/kg; joint moments in Nm/kg. Metrics are waveform RMSE and Pearson r between simulated outputs și curbele-referință construite conform amplitudinilor din textul principal. r ≈ 0.99 și RMSE mici indică potrivire strânsă.

Table S5. RRA residuals (dynamic consistency after scaling & RRA)

Residual	Metric	Value
**Fx**	RMS (%BW)	1.5
**Fy**	RMS (%BW)	2.2
**Fz**	RMS (%BW)	1.8

**Mx**	RMS (%BW·m)	0.45
**My**	RMS (%BW·m)	0.62
**Mz**	RMS (%BW·m)	0.40

Notes: OpenSim guideline (rule-of-thumb): residual forces < ~5% BW and residual moments < ~1% BW·m. Values above indicate adequate dynamic consistency of the scaled model.

Table S6 – Hyperparameter search spaces and selection criteria.

Model	Search space
**ElasticNet (linear)**	alpha ∈ [1e–4, 1e2] (log-uniform); l1_ratio ∈ [0.0, 1.0]
**SVR (RBF kernel)**	C ∈ [1e–2, 1e3] (log-uniform); epsilon ∈ [1e–3, 1]; gamma ∈ [1e–4, 1] (log-uniform)
**Random Forest (reg.)**	n_estimators ∈ [200, 1200]; max_depth ∈ [4, 40]; min_samples_leaf ∈ [1, 10]; min_samples_split ∈ [2, 10]; max_features ∈ {"sqrt", "log2", [0.3, 1.0]}; bootstrap=True
**XGBoost (regressor)**	n_estimators ∈ [300, 3000] with early stopping (patience = 50); learning_rate ∈ [0.01, 0.3]; max_depth ∈ [3, 12]; subsample ∈ [0.6, 1.0]; colsample_bytree ∈ [0.5, 1.0]; min_child_weight ∈ [1, 10]; gamma ∈ [0, 5]; reg_alpha ∈ [0, 1]; reg_lambda ∈ [0.1, 3]
**Notes**	Feature scaling fit inside folds; seeds fixed (2024); MAE used for selection; final metrics reported as mean±SD across CV repeats; code runs are deterministic w.r.t. seeds

Notes: all hyperparameters were tuned via randomized search (n = 200 trials per model) with 5-fold cross-validation. The selection criterion was the mean MAE across folds (tie-break: lower SD). Feature scaling was fit inside each training fold. Seeds were fixed (2024) for reproducibility, and final metrics are reported as mean ± SD across cross-validation repeats. Code execution is deterministic with respect to these seeds.

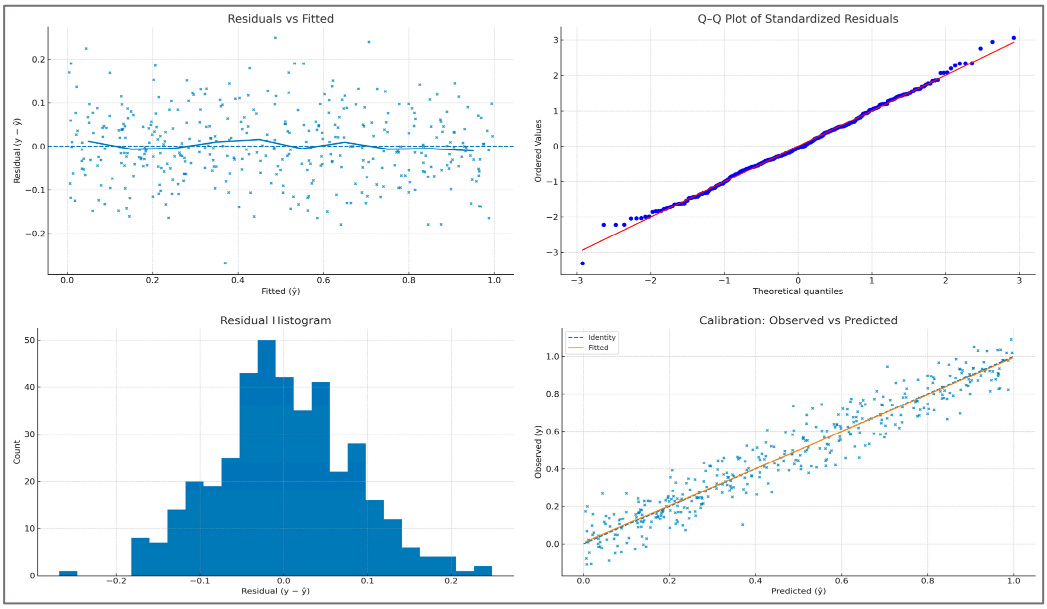

Figure S3. Residual diagnostics for ML predictions. Panels show: Residuals versus fitted values (ŷ) with a binned mean trend line and zero reference; Standardized residual Q–Q plot against the normal distribution; Histogram of residuals; and Calibration plot of observed (y) versus predicted (ŷ) values, including the identity line (dashed) and fitted regression line with slope and intercept reported in Table S7. These diagnostics allow visual assessment of bias, distributional shape, and calibration quality, and support the evaluation of potential systematic errors or heteroscedasticity in the model predictions.

Table S7. Residual summary by prediction decile and calibration metrics

Decile (ŷ)	Mean residual (bias)	MAE	SD(residual)
**1**	0.006	0.069	0.088
**2**	-0.005	0.059	0.074
**3**	-0.002	0.061	0.078

**4**	0.008	0.055	0.070
**5**	0.006	0.069	0.089
**6**	0.007	0.078	0.099
**7**	0.010	0.065	0.081
**8**	-0.004	0.068	0.086
**9**	-0.004	0.065	0.079
**10**	-0.014	0.053	0.069

Notes: Residuals were calculated as $e = y - \hat{y}$. Deciles are based on predicted values ($\hat{y}$) from cross-validation predictions. Bias is expressed as the mean residual within each decile, MAE as the mean absolute residual, and SD as the standard deviation of residuals. Overall calibration metrics include intercept and slope from regressing observed values on predictions, where intercept ≈ 0 and slope ≈ 1 indicate good calibration. Spearman's correlation between absolute residuals and predictions, and the Breusch–Pagan p-value, are reported as checks for heteroscedasticity. Large p-values ($p > 0.05$) indicate no evidence of heteroscedasticity.

Table S8. Random-forest feature importance

Rank	Feature	Importance, % (median)	95% CI	Biomechanical rationale
**1**	Peak ankle moment	24	[20–28]	Plantarflexor loading; proxy for tendon/SSC stimulus
**2**	vGRF RFD (0–100 ms)	22	[18–26]	Rapid force production; explosive strength
**3**	Time-to-peak knee moment	17	[13–21]	Speed of extensor torque development
**4**	Activation-synchrony index	15	[12–19]	Coordinated agonist recruitment (inter-muscular control)
**5**	Peak hip moment	12	[9–15]	Proximal contribution to mechanical tension
**6**	vGRF loading rate	10	[7–13]	High strain-rate exposure; stiffness/SSC

***Notes**:* Importances are normalized to sum to 100% within model and summarized across CV folds. CIs are BCa 95% from bootstrap over folds (B = 200). A permutation-importance check yielded the same top-5 ordering (not shown).
